# A composite index of avoidable hospitalization to assess primary care quality: an Italian experience

**DOI:** 10.1093/eurpub/ckac129.539

**Published:** 2022-10-25

**Authors:** G Damiani, M Cuomo, C Mencancini, A Burgio, A Solipaca, D Catania, A Heidar Alizadeh, P Arcaro, G Baglio

**Affiliations:** 1 Department of Life Sciences and Public Health, Università Cattolica del Sacro Cuore, Rome, Italy; 2 Department of Life Sciences and Public Health, Policlinico Gemelli Foundation, Rome, Italy; 3 Statistics and Health Information Flows Unit, National Agency for Regional Health Services, Rome, Italy; 4 Research, Statistics Department, Italian National Statistical Institute, Rome, Italy; 5 National Outcomes Evaluation Programme, National Agency for Regional Health Services, Rome, Italy

## Abstract

**Background:**

In Italy, primary care (PC) ensures universal health coverage while containing costs. However, the assessment of its quality still remains an issue. Evidence has shown that high-quality outpatient care, through timely interventions to prevent complications of “ambulatory care sensitive conditions”, may avoid hospitalization. Aim of the study is to analyse the performance of PC in the Italian regions, using a composite and synthetic index of avoidable hospitalizations.

**Methods:**

Hospital discharge data from 119 Italian geopolitical areas were analysed for the 2017-2019 triennium and for 2020, separately. According to the “Italian National Outcomes Evaluation Programme” methodology, 9 avoidable hospitalization indicators covering 5 nosological fields (infectious, respiratory, metabolic, cardiovascular and mental diseases) were combined in a synthetic index, calculated as the weighted mean of their standardized scores (with equal weights for each field). Using “natural breaks” technique, the areas were grouped into 5 clusters: “high”, “medium-high”, “medium”, “medium-low” and “low”.

**Results:**

The analysis showed a marked heterogeneity at intra-regional level for the pre-pandemic triennium, with areas of homogeneity in regions with higher levels of hospitalization. The “medium” cluster, which is the widest, included 36 areas variously distributed across regions. The comparison with 2020 confirmed the geographical patterns observed for the previous triennium, despite a general reduction in hospitalizations due to the pandemic.

**Conclusions:**

As a proxy indicator of PC quality, this index can aid decision makers in prioritizing quality improvement actions. However, in order to obtain a comprehensive evaluation, a joint reading of this index with other healthcare indicators is recommended.

**Key messages:**

• High-quality PC is essential in maintaining appropriate hospitalization levels.

• The composite synthetic index proposed could aid PC quality assessment.

